# Neural crest cell-derived pericytes act as pro-angiogenic cells in human neocortex development and gliomas

**DOI:** 10.1186/s12987-021-00242-7

**Published:** 2021-03-20

**Authors:** Francesco Girolamo, Ignazio de Trizio, Mariella Errede, Giovanna Longo, Antonio d’Amati, Daniela Virgintino

**Affiliations:** 1 Department of Basic Medical Sciences, Neuroscience and Sensory Organs, Human Anatomy and Histology Unit, University of Bari School of Medicine, Bari, Italy; 2 Intensive Care Unit, Department of Intensive Care, Regional Hospital of Lugano, Ente Ospedaliero Cantonale, Lugano, Switzerland; 3 Department of Basic Medical Sciences, Neuroscience and Sensory Organs, Molecular Biology Unit, University of Bari School of Medicine, Bari, Italy; 4 Department of Emergency and Organ Transplantation, Pathology Section, University of Bari School of Medicine, Bari, Italy

**Keywords:** Human brain development, Prosencephalon, Microvessels, Pericytes, Neural crest cells, Tunnelling nanotubes, Angiogenesis, Blood–brain barrier, Human gliomas

## Abstract

Central nervous system diseases involving the parenchymal microvessels are frequently associated with a ‘microvasculopathy’, which includes different levels of neurovascular unit (NVU) dysfunction, including blood–brain barrier alterations. To contribute to the understanding of NVU responses to pathological noxae, we have focused on one of its cellular components, the microvascular pericytes, highlighting unique features of brain pericytes with the aid of the analyses carried out during vascularization of human developing neocortex and in human gliomas. Thanks to their position, centred within the endothelial/glial partition of the vessel basal lamina and therefore inserted between endothelial cells and the perivascular and vessel-associated components (astrocytes, oligodendrocyte precursor cells (OPCs)/NG2-glia, microglia, macrophages, nerve terminals), pericytes fulfil a central role within the microvessel NVU. Indeed, at this critical site, pericytes have a number of direct and extracellular matrix molecule- and soluble factor-mediated functions, displaying marked phenotypical and functional heterogeneity and carrying out multitasking services. This pericytes heterogeneity is primarily linked to their position in specific tissue and organ microenvironments and, most importantly, to their ontogeny. During ontogenesis, pericyte subtypes belong to two main embryonic germ layers, mesoderm and (neuro)ectoderm, and are therefore expected to be found in organs ontogenetically different, nonetheless, pericytes of different origin may converge and colonize neighbouring areas of the same organ/apparatus. Here, we provide a brief overview of the unusual roles played by forebrain pericytes in the processes of angiogenesis and barriergenesis by virtue of their origin from midbrain neural crest stem cells. A better knowledge of the ontogenetic subpopulations may support the understanding of specific interactions and mechanisms involved in pericyte function/dysfunction, including normal and pathological angiogenesis, thereby offering an alternative perspective on cell subtype-specific therapeutic approaches.

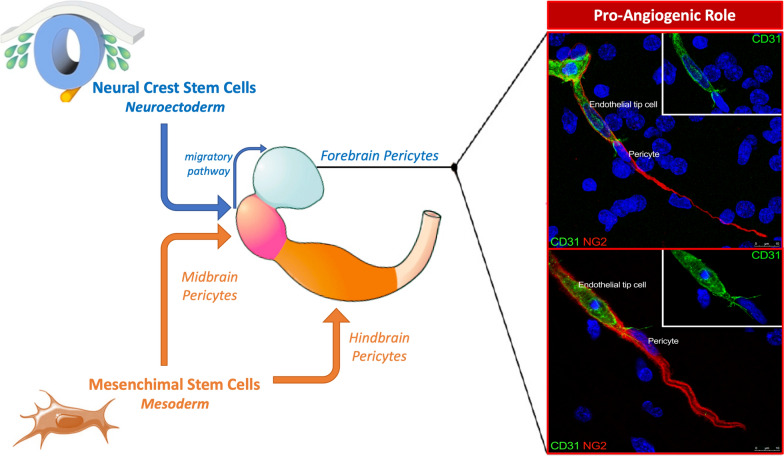

## Background

**Fig. 1:**
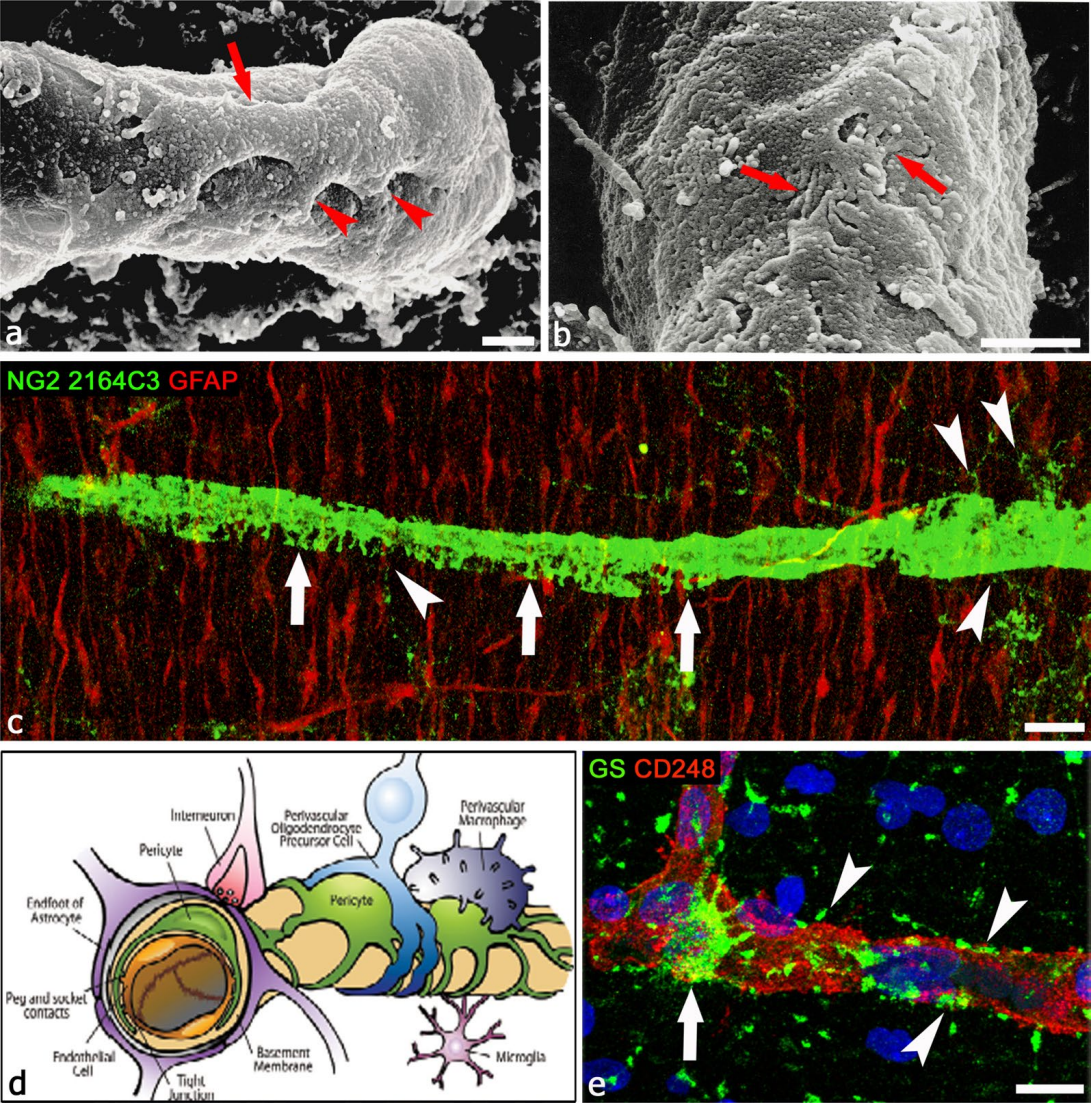
Pericyte morphology and relationships within the NVU. **a**, **b** Scanning electron microscope images of 14-day-old chick embryo microvessels, showing in **a** primary (red arrow) and secondary (red arrowheads) pericyte processes and in **b** their highly indented and interdigitated finger-like processes (red arrows) [from [5] with permission]. **c** Dorsal wall of the telencephalic vesicles (forebrain, future neocortex) of an 18-week-old human fetus, GFAP^+^ (glial fibrillary acidic protein) radial glia fibers and a pericyte coverage NG2 2164C3^+^, the latter shows finger-like processes (arrows); note the very fine perivascular processes of OPCs (arrowheads). **d** A schematic representation showing NVU components: ECs, PCs, perivascular astrocytes, vessel-associated microglial cells, OPCs/NG2-glia, macrophages, nerve fiber terminal [from [13] with permission]. PCs, embedded in the vessel basal lamina (here not shown) are the cells closest to the endothelium and display a variety of extensive contacts on their abluminal surface, in particular the relation with astrocytes and OPCs/NG2-glia [10]. **e** Astrocyte-pericyte relations are shown by glutamine synthetase (GS), confined within the astrocyte body (arrow) and in perivascular endfeet (arrowheads), most of which are in contact with CD248^+^ PCs rather than, directly, with ECs. Scale bars **a**, **b** 1 µm; **c** 20 µm; **e** 10 µm

**Fig. 2:**
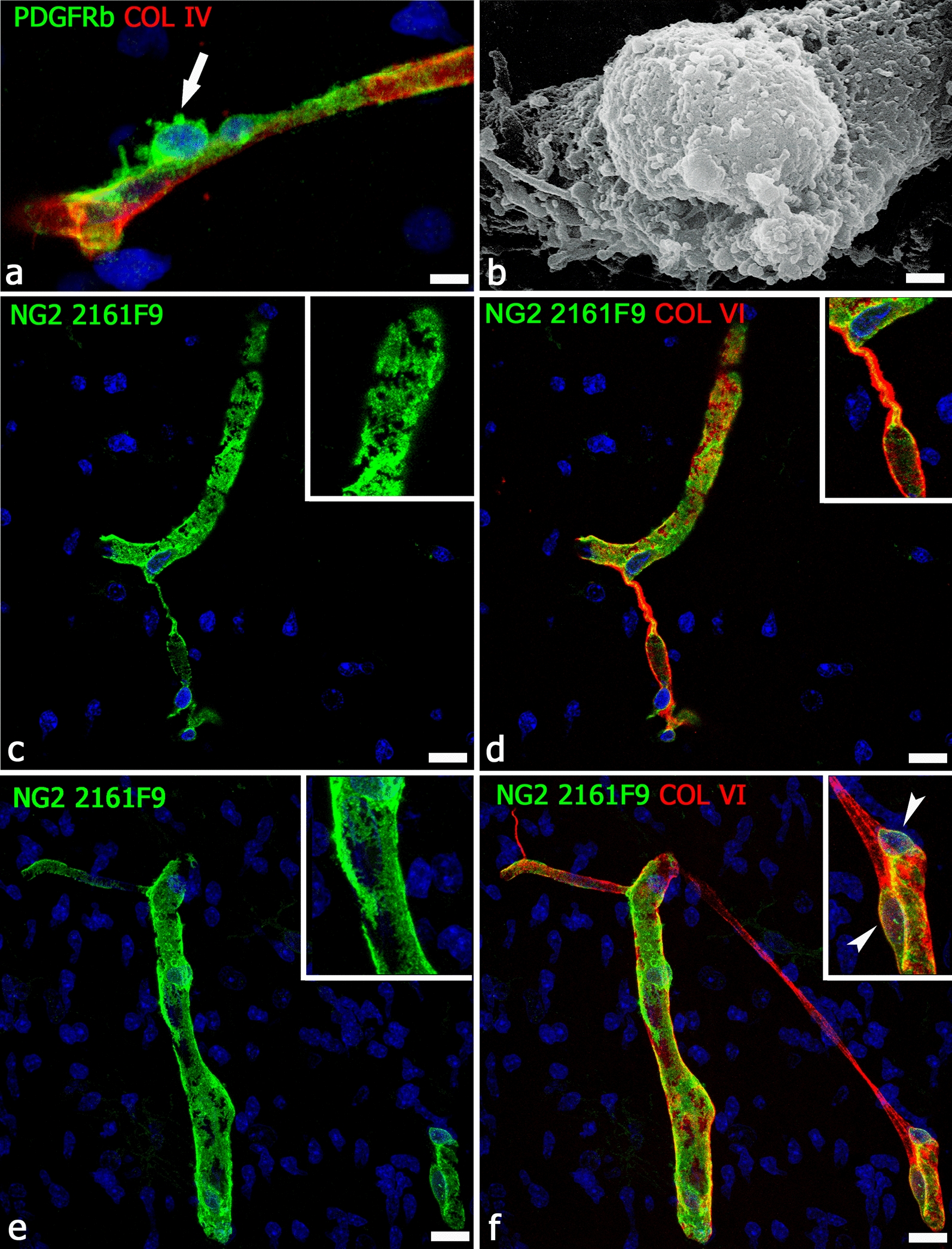
Pericyte morphology and vessel basal lamina relationships. **a** Morphology of an activated, PDGFR-β^+^ pericyte in contact with the collagen type IV^+^ basal lamina of a neocortex microvessel from a 22-week-old human fetus; note the abluminal bumpy surface of the PC (arrow), a detail well-depicted by the scanning electron microscopy 3D image (**b**; 14-day-old chick embryo) [from [5] with permission]. **c**, **d** The NG2 isoform, specifically recognized by antibody 2161F9, is able to outlines the finer cell details, thus describing the real extension of the pericyte coverage (**c**, inset) and its relation with the collagen VI-enriched basal lamina (**d**); note a pericyte conduit and its collagen VI sleeve (**d**, inset). **e**, **f** NG2 2161F9 immunostaining shows few large gaps in the pericyte coverage (better shown in **e**, inset); on the same field (**f**), a TNT/MT-like intervascular bridge is revealed by collagen VI staining; the inset shows two PCs close to the site of TNT/MT origin (arrowheads). **a**, **c**–**f**, Human telencephalon 22 wg. Scale bars **a** 7.5 µm; **b**–**f** 10 µm

The Rouget cells, firstly described by Charles-Marie Benjamin Rouget in the late 19th century [1], were later denoted as pericytes (PCs) [2]. They are described as vascular cells that, at the level of the microvessel segments (precapillary arterioles, capillary, and postcapillary venules) of the vascular tree, wrap around the endothelial cells (ECs), being retained within the vessel basal lamina, that is known to be formed by two layers, pertaining to ECs and astrocytes, respectively. Herein, the term ‘basal lamina’ is used instead of ‘basement membrane’, since brain microvessels only show a ‘basal lamina’ without the ‘lamina reticularis’ made up by fibrillar collagens, type I, III, and V. After the pioneering descriptions of brain PCs’ morphology in primates, including humans, gained by electron transmission microscopy (TEM) [3, 4], the emergence of scanning electron microscopy (SEM) has provided, together with subsequent 3D reconstructions by TEM serial sections [5, 6], a complete rendering of the 3D morphology and relationships of PCs (Figs. 1, 2). PCs show a prominent nuclear region bulging out on the abluminal vessel side, two longitudinally oriented primary processes sending out transversely arranged secondary processes and additional flat, finger-like, protrusions that interdigitate to fill the remaining gaps. As a consequence of their location within the neurovascular unit (NVU) of the central nervous system (CNS), PCs develop their two-sided activity: direct communication with ECs through peg socket connections and heterotypic gap junctions, interactions through extracellular matrix molecules and soluble factors, autocrine and paracrine signaling pathways, including those involved in the astrocyte-pericyte crosstalk [7, 8] and in interactions with all the other vessel-associated NVU components (Fig. 1) [9–13]. The NVU is essential in CNS homeostasis, neurovascular coupling, regulation of blood flow, as well as differentiation and functional activities of the blood-brain barrier (BBB) [14, 15]. In this context, PCs accomplish direct roles in leading microvessel development, maturation, and remodeling, finally stabilizing blood vessels and contributing to the BBB function [13, 16–24]. PCs, as the cells physically closest to the brain microvascular endothelium, also display immune activities characterized by the production of immune mediators such as nitric oxide and cytokines, thus participating in neuroinflammatory processes in brain infections and neurodegenerative diseases [13, 25, 26].

## Pluripotency and heterogeneity of pericytes

**Fig. 3:**
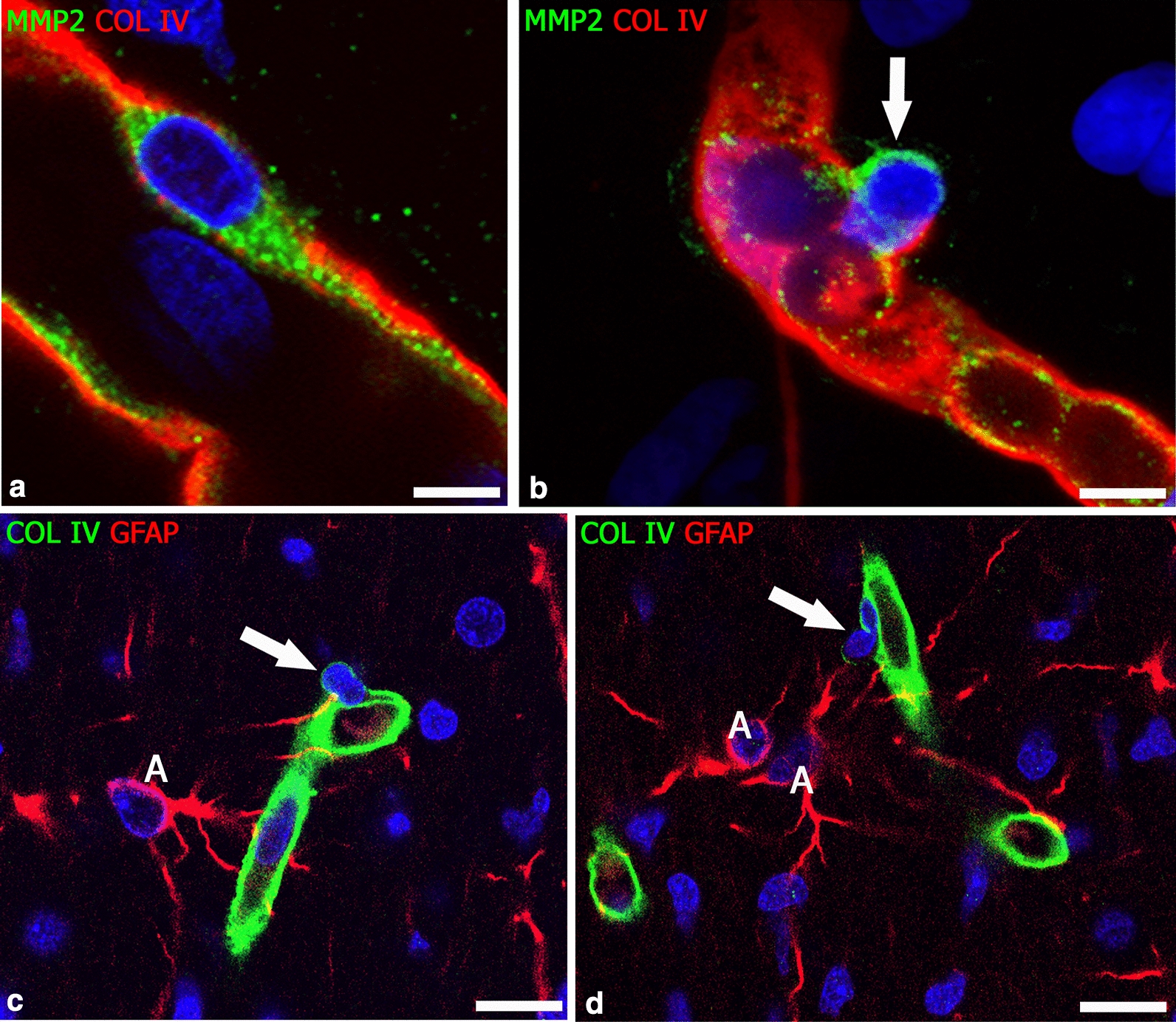
Resting and migrating PCs. **a** An MMP2^+^ resting pericyte embedded in the collagen IV vessel basal lamina and **b** a migrating pericyte in the act of breaking out by releasing enzyme MMP2 (arrow). **c**, **d** Active PCs (arrow) passing through the collagen IV-enriched basal lamina; note the trace of the enzymatically attenuated collagen IV. Human telencephalon 18 weeks of gestation. A, astrocyte. Scale bars **a**, **b** 7.5 µm; **c**, **d** 15 µm

The cell components pertaining to the NVU have recently been demonstrated to feature different levels of diversity, giving rise to the new concept of NVU heterogeneity [11]. Genome-wide association and RNA-seq studies have revealed morphological and functional astrocytes and microglia subtypes associated to both normal and pathological conditions [27–29]. Transcriptional profiling has highlighted the presence of different glial sub-populations [30–32], including neurotoxic, type A1, and neuroprotective, type A2, astrocytes associated to astrogliosis [33]. In addition, high-resolution transcriptomic analyses, together with the emergence of novel single-cell techniques and single-cell RNA sequencing, now propel studies of microglia heterogeneity, unveiling a variety of spatially and developmentally distinct microglia subtypes (for a Review see [34]. RNA-seq studies have also investigated PCs and their possible role in NVU heterogeneity [28, 35, 36]. Therefore, if it is correct to consider CNS PCs as motile, contractile cells, as proposed in Rouget’s original description [1], it is also true that heterogeneity and multitasking aptitude of PCs have already been pointed out [36–44]. Different subclasses of PCs along the capillary bed and in specific developmental and pathological conditions have been identified [13, 45]. These multiple profiles form the basis for the pericyte functional and phenotypic variety, including their differentiation along the mesenchymal lineage [46] (Table 1). PCs, as mesenchymal-like cells, are able to migrate by digesting the basal lamina molecules [41, 47–49] (Fig. 3) and to differentiate into fibroblasts [50–52], smooth muscle cells [53, 54], macrophages [55, 56], osteoblasts [57], myoblasts [58], adipocytes [59], chondrocytes [60], and also into neural and glial cells comprising oligodendrocyte progenitors [42, 61–65]. A general consensus holds that PCs are cells with a high plasticity, despite two studies challenged this concept [66, 67]. The response of PCs to specific cues in specific tissue contexts suggests that, in each of the vascular districts, PCs should be considered according to their origin and consequent morphological and functional singularities [25, 37, 68–71]. Accordingly, the variety of different pericyte subtypes [60, 72, 73] (Table 1) and the complexity of the PCs biology and genetic profile emerge, together with the variety of the pericyte-expressed molecules studies conducted up to now and the attempts at identifying specific pan-pericyte markers [42, 68, 74] (Table 2).

**Table 1:** Pericyte subpopulations according to ontogeny

Origin	Position	Gene expression	Roles
Neuroectoderm ⇓ Neural crest stem cells ⇓ Ectomesenchyme ⇓ Ectomesenchyme-derived pericytes	Forebrain Leptomeninges Forebrain vessels Retinal vessels Skull, face, neck tissues Truncus arteriosus Mesentery (?) [69, 75–81]	PAX3, PAX7, TFAP2A [82] FOXC1, FOXC2 [83]	Vessel development [76, 84–87] Glioblastoma neo-vessels [87–94]
Intraembryonic Mesoderm ⇓ Lateral mesoderm (mesothelium) ⇓ Mesenchyme ⇓ Mesenchyme-derived type 1 pericytes	Lung Heart Liver Gut [69, 78, 81, 95–98]	MIXL1, TBXT [82]	Absence in tumor vessels [99] Fibrosis, myopathies [58, 81]
Intraembryonic mesoderm ⇓ Paraxial mesoderm (sclerotome) ⇓ Mesenchyme ⇓ Mesenchyme-derived type 2 pericytes	Midbrain Hindbrain Spinal cord [69, 76, 78, 100, 101]	MIXL1, TBXT [82]	Vessel development Tumor neo-vessels Glioma neo-vessels [102, 103]
Extraembryonic mesoderm ⇓ Mesenchyme ⇓ Yolk sac-derived myeloid progenitor ⇓ Macrophage-derived pericytes	Midbrain Rostral back skin Retina [104, 105]	Kcnj8, Rgs5, Dlk1, and Abcc9, TGFBR2 [105]	Vascular anastomosis [106] Retinal vascular density [107] Tumor angiogenesis [108]

**Table 2:** Markers of pericytes expressed in healthy and diseased CNS (with BBB dysfunction)

Pericyte marker	Healthy CNS	Diseased CNS
Neuron-glial antigen 2 (NG2)	Development [10, 40, 80, 84, 88, 109–112]	Dementia [113] Healing wounds [114] Neurofibromatosis [115]. Neuroinflammation [116, 117] Tumor neovasculature [10, 87, 88, 114, 118–122]. Traumatic injury [123, 124]
NG2 isoforms	Development [88, 125]	Glioblastoma [88, 126] Ullrich’s congenital muscular dystrophy [125]
Platelet derived growth factor receptor beta (PDGFRβ)	Development [84, 127, 128] Adult [10, 18, 40, 70, 108, 110, 129–132]	Alzheimer’s disease [129, 133, 134] Amyotrophic lateral sclerosis [135, 136] Angiopathies [132, 137–142] Neuroinflammation [10, 123, 143, 144] Tumor neovasculature [108, 145]
Alanyl aminopeptidase (CD13)	Adult [10, 35, 70, 74, 128, 131, 146–149]	Neuroinflammation [150] Stroke [151]
Vimentin (VIM)	Adult [39, 131, 152, 153]	Angiopathies [154–156]
Regulator of G protein signaling 5 (RGS5)	Development [157–161]	Huntington’s disease [162] Stroke [163–166]
Smooth Muscle *α*-Actin (*α*-SMA)	Adult (pre- and post-capillary pericytes) [70, 128, 131, 167–172]	Retinal angiopathy [170] Familial form of Alzheimer’s disease [173]
Vascular endothelial growth factor (VEFG)	Development [174, 175]	Angiopathies [176, 177] Neurotoxicity [178]
CXCR4	Development [179–181]	Glioma [182, 183] Neuroinflammation [184]
Toll-like receptor 4 (TLR4)	Adult (transcriptome analysis) [185]	Stroke [186, 187]
ATP binding cassette subfamily C member 9 (ABCC9)	Adult [131, 188]	Aging [189]
Melanoma Cell Adhesion Molecule (CD146)	Development [74, 87, 190, 191]	Glioblastoma [87]
Vascular cell adhesion molecule-1 (VCAM-1)	FACS [192]	Neuroinflammation [56] Tumorigenesis [193]
Intercellular adhesion molecule-1 (ICAM-1)	FACS [192] Cell cultures [56]	Neuroinflammation [56, 194]
3G5-defined ganglioside	Adult [195]	Retinopathies [196, 197]
Angiopoietin 1 and 2 and Tie2 receptor	Development [198–201]	Diabetic retinopathy [202] Neurotoxicity [175] Stroke [203, 204]
Leptin receptor (LepRb)	Development [205]	Neuroinflammation [206]
Endosialin (CD248)	Development [84, 207–209]	Glioma [88, 208, 209]
Sphingosine-1-phosphate receptor 2 and 3 (S1PR2 and 3)	Adult [198, 210, 211]	Stroke [211, 212] Traumatic injury [213]
Transforming growth factor β (TGF β)	Adult [198] Cell cultures [171, 214]	Neuroinflammation [10, 215, 216]
Angiotensin 1 and 2 receptors (AT1 and AT2)	Cell cultures [179, 217, 218]	Diabetic retinopathy [219, 220]
ATP-gated Purinergic 2X receptor cation channel (P2X7R)	Adult [221]	Diabetic retinopathy [222] Neuroinflammation [221, 223]
Zic1	Development [83]	
Potassium inwardly-rectifying channel (Kir6.1)	Adult [131, 188, 224]	
Delta Like Non-Canonical Notch Ligand 1 (DLK1)	Adult (microarray analysis) [188]	
Vitronectin (VTN)	Development [110, 225]	
Interferon-induced transmembrane protein 1 (Ifitm-1)	Development (transcriptome analysis) [110]	
Myosin light chain phosphatase (MLCP)	Cell culture [226]	
Fluoro-Nissl dye NeuroTrace 500/525	Adult [227]	
Forkhead transcription factor C1 (FoxfC1)	Development [83]	
Interferon-induced transmembrane protein 1(Ifitm-1)	Development (transcriptome analysis) [110]	
Connexin 30 (Cx30)	Adult [228]	
P-type ATPase (*Atp13a5gene)*	Adult (transcriptome analysis) [131]	
Basic fibroblast growth factor (bFGF)		Stroke [229]
Sox2 and Klf4		Stroke [230]
protein encoded by the *NOTCH3* gene		CADASIL angiopathy [231–234]
Bone morphogenetic protein 4		Alzheimer’s disease, angiopathies [235]

### Neural crest cells and head morphogenesis

Wilhelm His, observing the CNS development in neurula-stage chick embryos, was the first to describe the appearance of neural crest cells (NCCs) (Zwischenstrang) as cellular elements derived, but distinct, from the neuroectodermal cells that form the neuroepithelium of the neural tube [236]. Pioneering studies in fish demonstrated the capacity of these (neuro)ectodermal cells to colonize the embryo head [237]. However, despite of these early observations, the existence, distribution, and fate of the NCCs remained largely ignored by embryologists for decades, and then became the subject of active controversies. NCCs, soon after their detachment from the neuroectoderm fold lips, undergo an epithelial-to-mesenchymal transition, becoming hardly distinguishable, along their migratory pathways and inside the colonized tissues and organs, from typical mesenchymal cells of mesodermal origin. It was an embryologist, Julia Platt [238], who first recognized the head mesenchyme as derived from NCCs and coined the term ‘mesectoderm’ to denote the mesenchyme of neuroectodermal origin (now known as ‘ectomesenchyme’), distinct from the ‘mesentoderm’, a term that indicated the mesenchyme which originates from the mesodermal germ layer (now simply ‘mesenchyme’). More recently, after more than half a century from these observations, the role of NCCs during head morphogenesis began to be unveiled by fate-mapping experiments [239]. Subsequently, embryo-to-embryo transplant studies in the chick-quail chimera experimental models made it possible to define the NCCs as a pluripotent, ‘stem’, embryonic cell population (neural crest stem cells, NCSCs), able to develop into a large variety of tissues, including cartilages, membranous bones, cartilaginous bones and other connective components, such as dermis and tendons, and also skeletal and visceral muscles, during skull (neurocranium) and face- (splanchnocranium) and neck-branchial regions development [240–244]. In addition, the NCSC-derived ectomesenchyme gives origin to the leptomeninges, including the forebrain leptomeninges, and is necessary for neuroepithelium survival and vascularization [239, 240, 245] (Table 1).

### Neural crest stem cell-derived pericytes

Little is known about the exact identity of pericyte ancestors within developing tissues, and distinct developmental sources have been demonstrated, highlighting that the embryonic origin of PCs differs among tissues and organs [69, 246, 247]. Several studies using lineage tracing methods indicate that PCs in part of the cephalic region and thymus have an ectomesenchyme origin [248–252], while in the lung, heart, liver and gut, PCs derive from the mesothelium. Thus, they have a lateral mesoderm, epithelial-like, mesenchymal origin [69, 78, 95–98]. In most other organs, PCs derive from the paraxial mesoderm, specifically the sclerotome compartment, so again they have a mesenchyme origin [69, 76, 78, 100] (Table 1).

### Neural crest stem cell-derived forebrain pericytes

During embryonic neurogenesis, NCSCs are concentrated at the cranial and ventral secondary encephalic vesicles (telencephalon and diencephalon) of the forebrain. In this region, unlike in the remaining parts of the brain (midbrain, hindbrain) [253, 254], PCs, hereafter named forebrain PCs, derive entirely from NCSCs, thus they represent a subset of PCs with a specific ontogeny and are distally sharply delimited by the midbrain [69, 75–80]. In the anterior/ventral head regions, NCSCs are initially present in the ectomesenchymal layer comprised between the surface ectoderm and the developing CNS, where they differentiate into PCs and become associated with mesoderm-derived endothelial precursors that express VEGFR2 (vascular endothelial growth factor receptor 2) [76]. The resulting vascular plexus then ramifies and vascularizes the forebrain leptomeninges (arachnoid mater and pia mater), retinal choroids, and facial structures. Therefore, as already described, NCSCs participate in the constitution of the forebrain meninges [239, 240], which enclose the deeper, pial capillary network, necessary for later vascularization of the brain. Passing through the meninges, capillaries with PCs of ectomesenchyme origin supply the forebrain, while capillaries with PCs of mesenchyme origin supply the mesencephalon, the rhombencephalon and the spinal cord. An intriguing aspect of PCs origin and heterogeneity is the demonstration of PCs localized in the mouse embryonic rostral back skin, an ectodermal derivative, and some PCs in the midbrain, a neuroectodermal derivative, sharing the same origin with myeloid progenitors; these cells differentiate into PCs under the TGF-β (transforming growth factor-β) signaling control [104, 105].

### Generation of pericytes by hiPSC-derived neural crest cells

Mesoderm-derived PCs and NCC-derived PCs can be obtained from induced pluripotent stem cell (iPSC) [77, 82, 255]. A recent study [82], starting from human iPSC obtained from healthy and AD patients (human iPSC; hiPSC), developed two differentiation-inducing protocols serving to generate both mesoderm-derived (mesenchymal) PCs and NCC-derived (ectomesenchymal) PCs. Firstly, hiPSCs were grown in either a mesodermal induction medium or in neural crest induction medium, in order to generate mesodermal cells and NCCs, respectively. Following induction, cells were passaged and maintained in pericytes medium, which stimulates pericytes differentiation. The pericyte identity of both mesoderm- and NCC-derived PCs was demonstrated by the expression of pericyte cell-surface markers, PDGFR-β (platelet-derived growth factor receptor-β), NG2 (neuron-glial antigen 2), CD13 and CD146, and of brain pericyte-specific genes, vitronectin and the forkhead transcription factors, FOXF2 and FOXC1. Interestingly, FOXF2, which is expressed by NCCs during development, was primarily expressed by NCC-derived PCs, while WNT signaling seemed to be specifically associated to pericyte development through the NCC pathway. Reliable methods for engineering brain-specific subpopulations of PCs from hiPSCs are a promising improvement of in vitro studies on both barriergenesis and angiogenesis. However, the main limitation for iPSCs derived PCs and others NVU cell components remains the lack of the important contribution of cell–cell contact and fluid shear stress and, moreover, the maturation of these cells to the adult brain PCs. The roles of major signaling pathways on them and their secretome have not been studied yet [256]. Nonetheless nowadays stem cell-based BBB models represent the main tool for neurodegenerative, neuroinflammatory and brain tumor disease modeling where PCs may play important underestimated roles.

## Human neocortex and the developing NVU

In the entire CNS, within the NVU, PCs are heavily involved in maintaining tissue homeostasis, vessel stability, and integrity of BBB cellular and molecular mechanisms [257–270]. Nonetheless, specific properties have been observed for NCSC-derived PCs, that contribute to the vascularization of forebrain that will develop the telencephalon dorsal wall (future neocortex), where the origin of forebrain PCs from NCSCs seems to entail additional biological functions, involved in both angiogenesis and barriergenesis [271, 272]. In our studies on human telencephalon development and vascularization, we have relied on the detection of NG2, an integral membrane chondroitin sulphate proteoglycan encoded by the Cspg4 gene pericyte marker (Fig. 2). NG2 was firstly identified as an important neural cell surface antigen by Stallcup and Cohn [273] and its expression by active, immature PCs and proliferating oligodendrocyte precursor cells (OPCs) was demonstrated [274, 275]. The large juxtamembrane extracellular domain (D3) of NG2 mediates several cell–cell and cell–matrix interactions, including a fundamental role in endothelial cell adhesion and spreading (for a comprehensive review please see Nishiyama et al. [276]).

### The forebrain pericytes leading role in human cerebral cortex vascularization

**Fig. 4:**
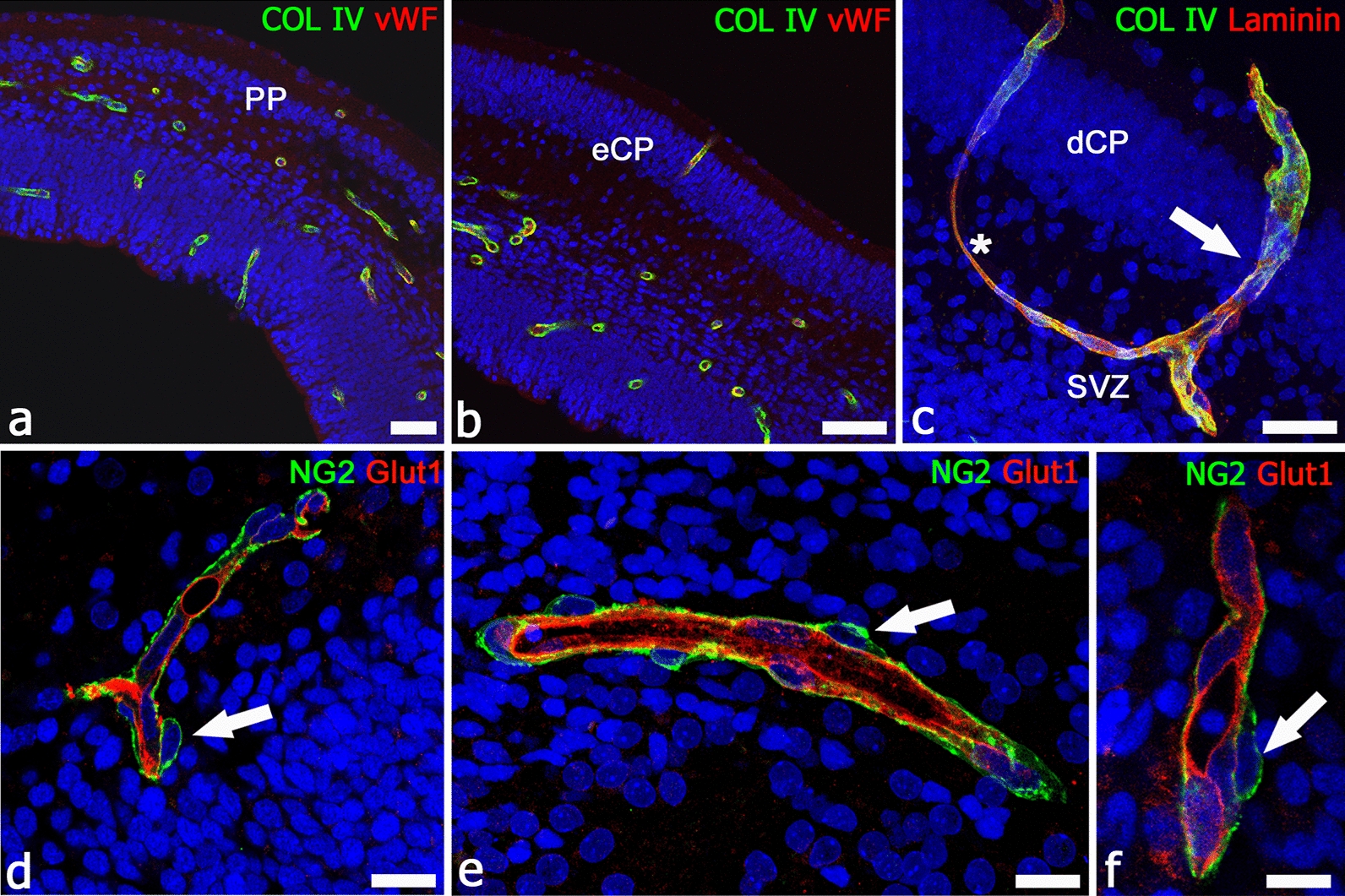
First steps in human dorsal telencephalon vascularization. **a**–**c** Sequence of cerebral cortex formation and vascularization at 9/9.5 weeks of gestation (**a**, pre-plate; PP), 10 weeks of gestation (**b**, early cortical plate; eCP), and 12 weeks of gestation (**c**, developing cortical plate; dCP): the newly penetrated microvessels are lined by von Willebrand factor (vWF)-reactive ECs and surrounded by collagen IV (**a**, **b**) and by collagen IV and laminin (**c**); note in **c** a penetrating microvessel (arrow) that branches in the subventricular zone (SVZ) and forms a loop-like anastomosis (asterisk). **d**–**f** During these early phases of cerebral cortex vascularization, ECs express the BBB-specific transporter Glut1 and are enwrapped by a continuous layer of NG2^+^ PCs (arrow). **d**–**f** Human telencephalon 12 weeks of gestation. Scale bars **a** 40 µm; **b** 10 µm

**Fig. 5:**
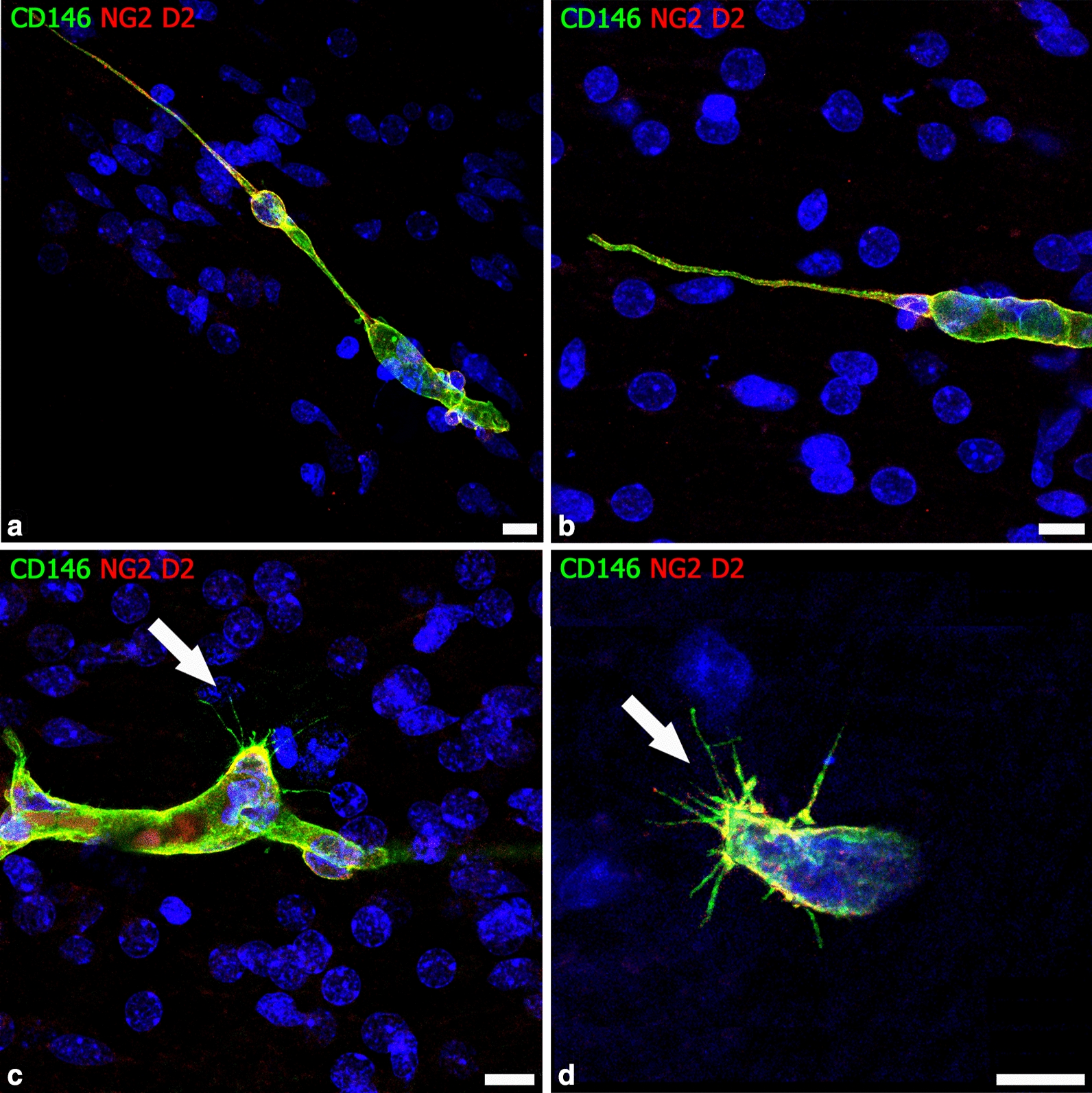
Pericyte-derived leading structures during human cerebral cortex vascularization. **a**, **b** Forebrain PCs, revealed by colocalization of NG2 and CD146, form the leading tip of cerebral cortex growing microvessels and give rise to TNT-like (**a**) and MT-like (**b**) structures. **c**, **d** CD146 staining unveils the filopodial processes of NG2^+^/CD146^+^ sprouting PCs (arrow). (**a** from [87] with permission). Human telencephalon 22 weeks of gestation. Scale bars **a**–**c** 10 µm; **d** 7.5 µm

**Fig. 6:**
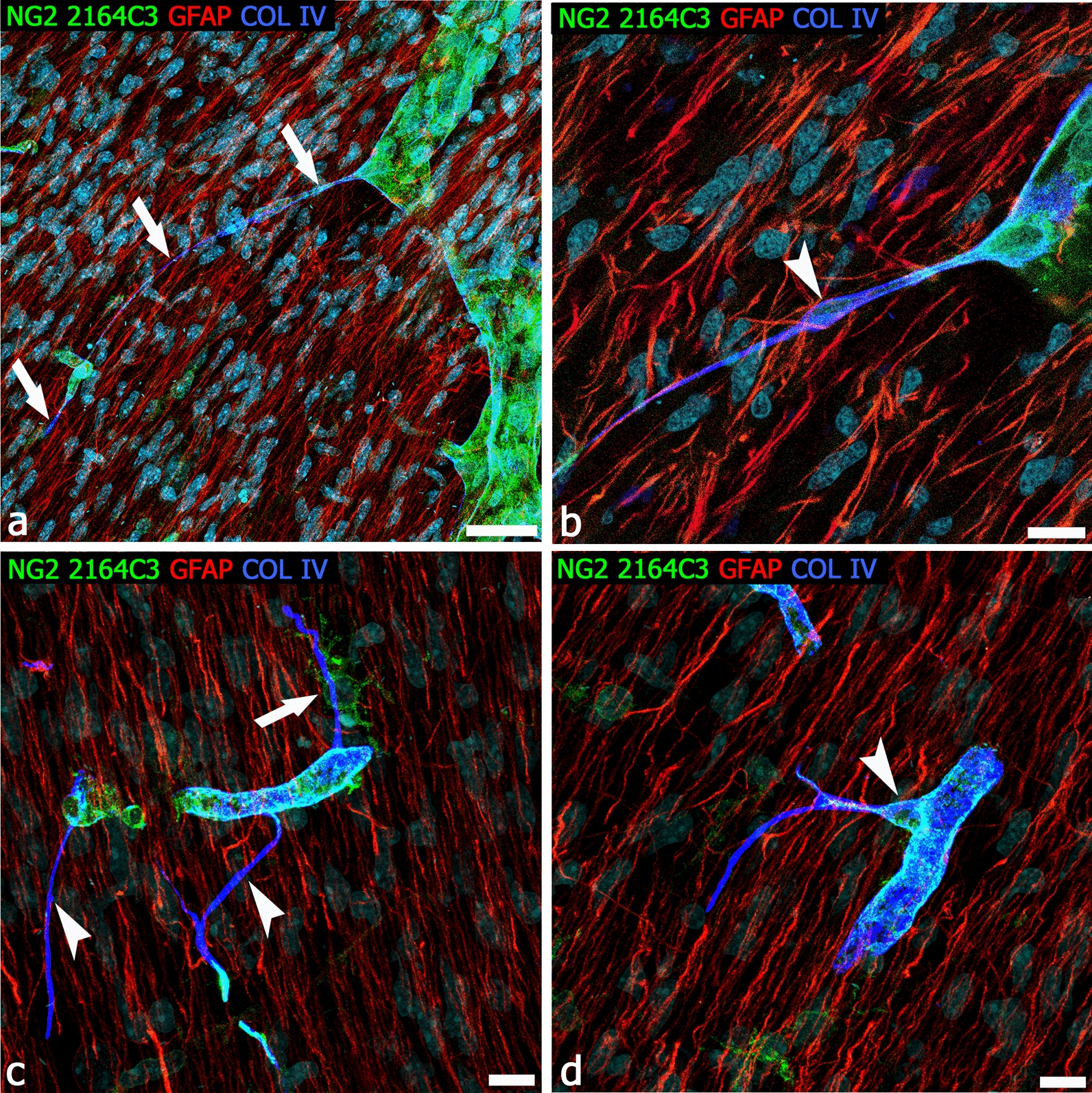
Radial glia/pericyte TNT relationships in the human developing cerebral cortex. **a**, **b** Triple staining with antibody NG2 2164C3, GFAP, and collagen IV reveals a very long pericyte TNT and the accompanying collagen IV basal lamina (**a**, arrows), enlarged on a single optical plane in **b**; note a TNT conveyed nucleus (arrowhead) and the extensive relations with GFAP^+^ radial glia fibers. **c** Multiple NG2^+^collagen IV^+^ TNTs (arrowheads) arise from the same parental vessel, one of which receives multiple contacts from a perivascular NG2^+^ OPC (arrow). **d** A ramified TNT arises from the pericyte body (arrowhead*).* Human telencephalon 22 weeks of gestation. Scale bars **a** 20 µm; **b**–**d** 10 µm

**Fig. 7:**
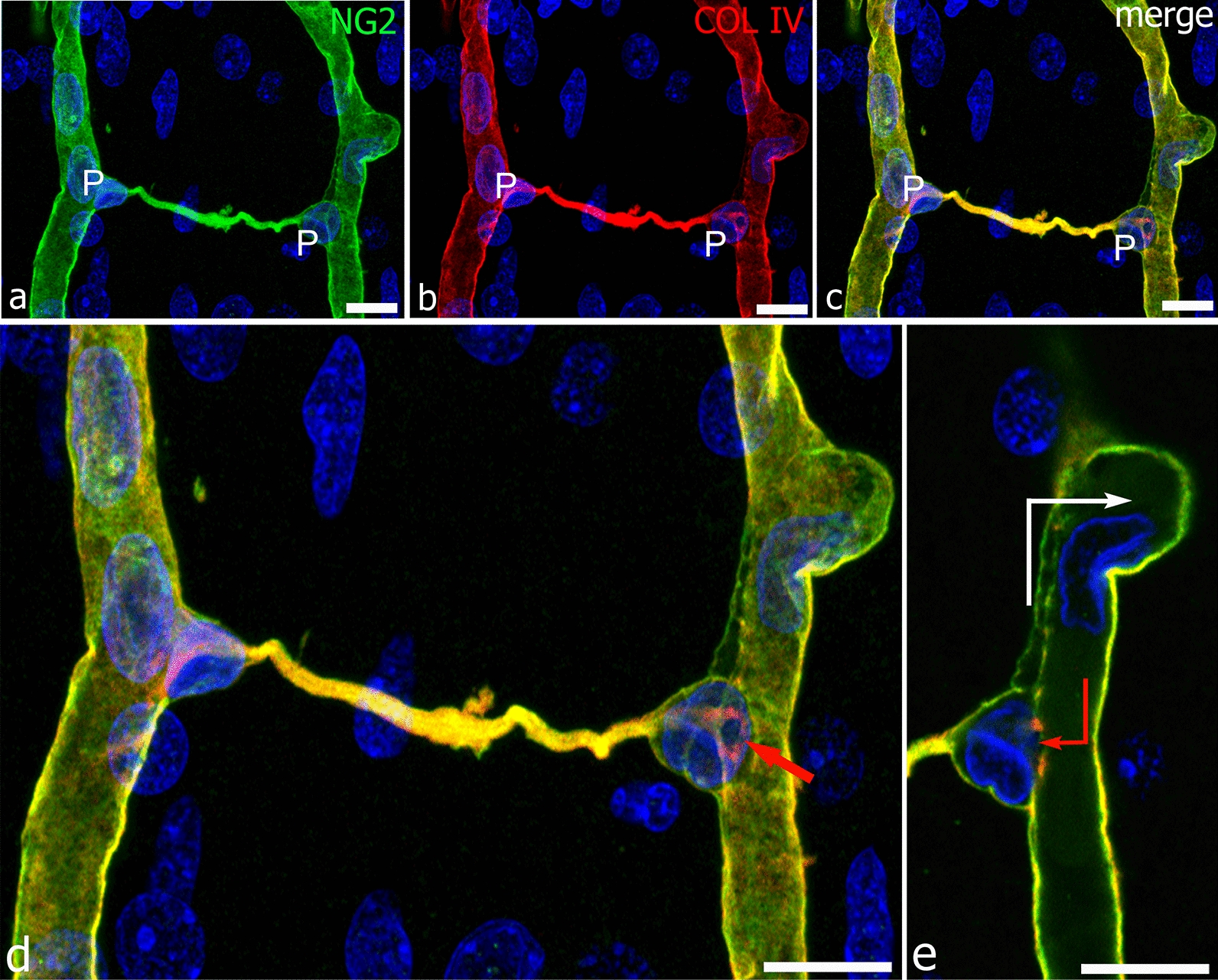
A pericyte conduit between facing radial vessels of the developing human cerebral cortex. **a**–**c** Two pericytes (P) are located at the opposite terminals of an NG2^+^collagen IV^+^ bridging conduit; as often observed, their nucleus marks the point of TNT/MT origin. **d** The enlargement of the merged image in **c** reveals further details and shows that in both the PCs, the nucleus is bent over on itself, describing a phrygian hat-like shape, so leaving an opening directly communicating with the lumen of the parental vessel; the entrance to the ‘tunnel’ is revealed by the collagen IV-enriched endothelial layer of the vessel basal lamina (red arrow). This critical passage is better shown in the single optical plane from the z-stack (**e**, red arrow); note the nucleus of an EC (white arrow) engaged through a collateral root. Human telencephalon 22 weeks of gestation. Scale bars **a**-**e** 25 µm

In humans, a large part of organogenesis (early ontogenesis) takes place during the embryonic period, that is limited to the first 8 weeks of embryonic development, while ontogenesis will continue during the subsequent fetal development. At the 9th week of gestation [277, 278], the telencephalic vesicles are already surrounded by a perineuronal vascular plexus of a composite origin: mesenchyme-derived ECs and ectomesenchyme-derived PCs [76], in fact, NCCs give origin to the PCs, although not to the ECs [240, 279]. When the cerebral cortex starts to form, soon after the pre-plate stage (9–9.5 weeks of gestation), vessel sprouts originate from the perineural vascular plexus and, guided by a VEGF gradient [127, 280], radially invade the nervous wall, elongate, and start to branch at their distal ends [281–284] (Fig. 4). Therefore, NCC-derived PCs associated with these parenchymal microvessels, including those associated to the vascular bed of the choroid plexuses [76], are already present at the very beginning of brain vascularization. In human developing cortex, NG2^+^ forebrain PCs are promptly detectable, together with early NVU radial glia components [84, 85] and with EC structural and functional hallmarks of BBB differentiation (Fig. 4). In fact, in humans the process of cerebral cortex vascularization seems to proceed in parallel with the appearance of an endothelial BBB phenotype and barrier devices, such as endothelial tight junctions [285], metabolic transporters [286], and efflux transporters [287]. This distinctive feature highlights the vital role played by the BBB also during CNS development, as recently confirmed by an in vivo study on transgenic zebrafish lines [288]. Human forebrain PCs that establish tight relations with ECs during the earliest stages of vessel growth [84], and contribute to vessel stability [51] and BBB function [40], also appear to play important roles during angiogenesis and vessel branching. In fact, forebrain PCs, identified by NG2 and CD146, have been observed at the leading edge of growing vessels [289], where these cells are able to raise tunnelling nanotubes (TNTs) and microtubes (MTs) and, like ECs, are also seen to form leading sprout-like structures (Fig. 5) [87]. Pro-angiogenic PCs, surrounded by a collagen type IV- and type VI-enriched basal lamina, appeared always in contact with radial glia cells (Fig. 6) [87]. Pericyte MTs have been described as EC-free conduits [89], then able to recruit ECs according to a process that seems to reverse the classical EC/pericyte interplay and that has been suggested as an alternative mode of vessel growth [84] (Fig. 7). These data, diverging from the classical angiogenic model consisting of endothelial sprouting and pericyte recruitment events [69, 127, 290], should be considered to reveal a direct angiogenic activity of PCs [291] and offer a possible ‘additional’ perspective on angiogenic mechanisms (Additional file 1: Figure S1). Pericyte TNT/MT-like structures, and a direct involvement of these cells in early angiogenesis, were firstly reported by Nehls et al. [292], who detected cord-like structures in whole-mount preparations of rat mesentery, composed solely of PCs at the sprouting front. The PCs lay at and in front of the advancing tips of endothelial sprouts and also bridged the gap between the leading edges of opposing endothelial sprouts. These observations mirror the description of pericyte TNT/MT as guiding structures aiding the outgrowth of ECs during human cerebral cortex vascularization [87]. Previous studies postulate an alternative contribution of PCs to neovascularization, describing endothelium-free pericyte tubes and segments of growing sprouts formed by PCs in both normally developing microvasculature of mouse retina and tumor vascularization (including melanomas and gliomas) [89], in murine tumor models [86], in subcutaneous matrigel plug assays, and in adult mouse cornea [293]. Interestingly, tubular structures, observed in tumors and denoted tumor microtubes (TMs), have been considered closely related to TNTs/MTs, although they possibly also have other functions [294]. It is therefore conceivable that conduit-forming PCs may be able to promote a self-regulated process of endothelization/lumenalization, through trans-basal membrane interactions [52], including the processes more directly mediated by NG2. In fact, ECs adhere to and spread on NG2-coated surfaces, and NG2 stimulates the migration of ECs and promotes corneal angiogenesis [295].

### The supportive paracrine role of pericytes

Besides the stabilizing role exerted by PCs on ECs [52, 158], there is an active angiogenic effect of PCs in secreting pro-regenerative molecules in response to PDGF-B [295, 296]. Of particular note is VEGF, which has been immunolocalized in PCs during human cerebral cortex development [174] and is released by these cells in in vitro models [175, 296, 297]. In a mathematical, biomimetic 3D angiogenesis model, it has been demonstrated that PCs intervene in the VEGF/TNF-α (tumor necrosis factor-α) proangiogenic/antiangiogenic interplay, promoting a proangiogenic effect of TNF-α, thus allowing complete VEGF-induced sprout formation, elongation, and lumenalization, and also ensuring that the efficacy of the reverted TNF-α effect is proportional to the extension of the pericyte coverage. In fact, TNF-α activity is fully inhibitory with a very low pericyte coverage, and switches sharply to strongly proangiogenic in the presence of a uniform pericyte coverage [298]. In the above-cited study on mesoderm- and NCC-derived PCs obtained from induced pluripotent stem cells (hiPSCs) [82], it was demonstrated that both mesoderm- and NCC-derived PCs are able to induce the formation of endothelial lumenalized tube-like structures and that the activity of NCC-derived PCs was significantly more effective (Additional file 2: Figure S2).

### The forebrain pericytes leading role in glioblastoma neo-angiogenesis

**Fig. 8:**
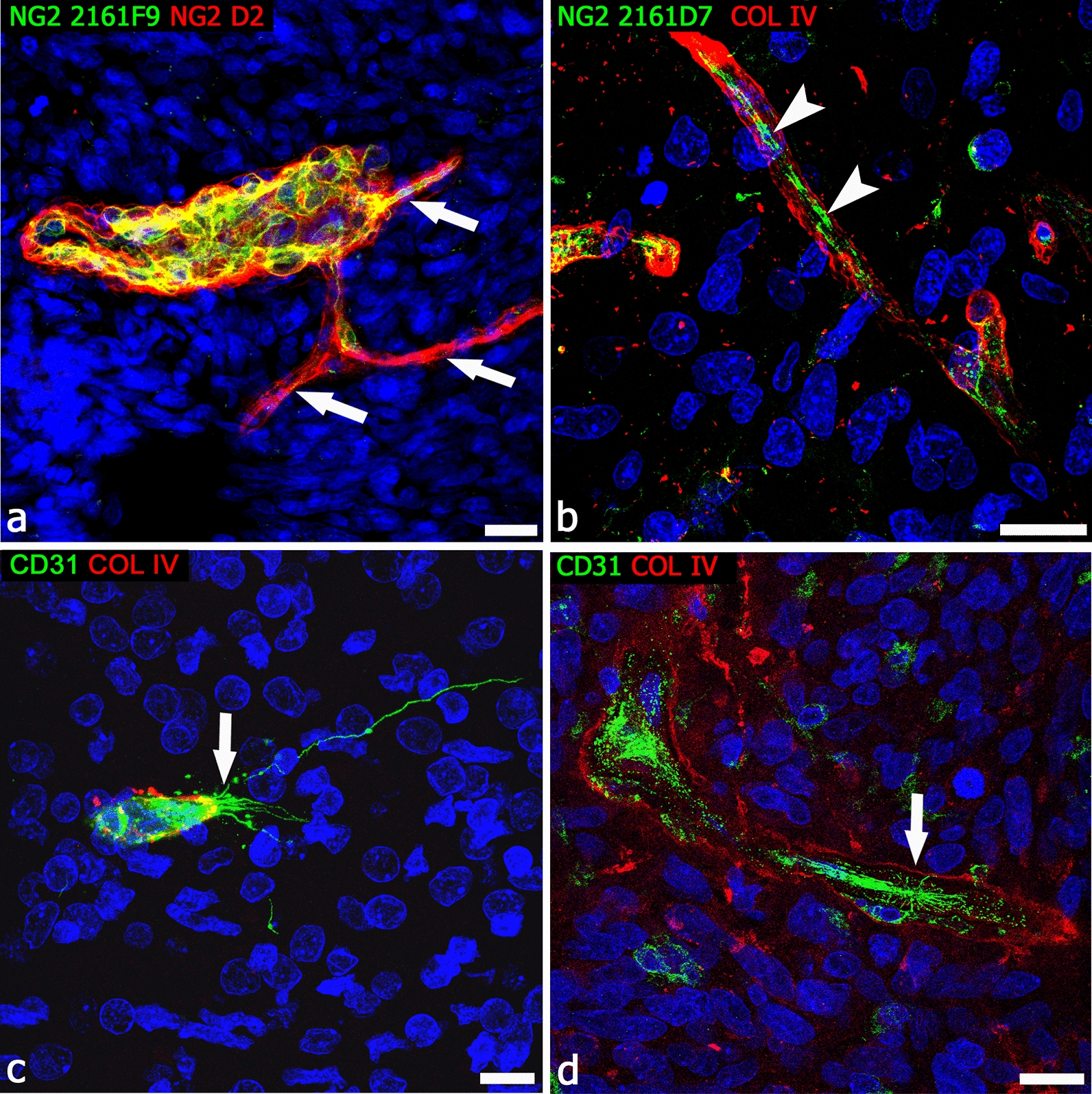
Example of alternative modes of tumor vessel growth in human GBM. **a**, **b** Multiple, EC-free pericyte conduits arise from a tumor vessel characterized by multilayers of PCs labeled by different NG2 isoforms (**a**, arrows) and an NG2^+^ pericyte MT surrounded by the collagen IV basal lamina (**b**, arrowheads). **c** A typical vessel sprout observed during cerebral cortex vascularization in a human fetus at 22 weeks of gestation; the CD31^+^ endothelial tip cell is characterized by a TNT-like process (arrow), a number of shorter, exploring filopodia, and a cloud of tip cell-associated microvescicles, confront with a GBM mimicking vessel sprout (**d**, arrow) formed by CD31^+^ glioblastoma cell-derived ECs [90, 92], surrounded by a disassembled collagen IV basal lamina and numerous, scattered, CD31^+^ cells. This growing structure closely resembles glioblastoma cells described migration in vitro through a 3D matrix [91]. Scale bars **a**, **b** 20 µm; **c**, **d** 10 µm

In our hypothesis, forebrain PCs may display a unique angiogenic aptitude as compared to the PCs of mesodermal origin, found in other regions of the CNS. Exploratory studies of pericyte-endothelial relationships during human fetal brain vascularization revealed an intimate interplay between the ECs and the leading activity of forebrain PCs in vessel sprouting events [84, 289]. Notably, glioblastoma multiforme (GBM) is the most highly vascularized brain neoplasm, it is characterized by very active and diverse angiogenic mechanisms, and by a tumor microvascular architecture heterogeneity, including tumoral cell channels (vessel mimicry), intussusceptive vessels, and glomeruloid vessels [299, 300]. In GBM, we have observed the presence of several glomeruloid vessels, where NG2+/CD248+ PCs, expressing a variety of NG2 molecular forms, proliferate and form a multilayered shell [88]. Hyperplastic PCs, whose rate of proliferation increases with the glioma grade, but not ECs, that appear confined to the monolayer lining cells, have been described as the main feature of higher grade glioma vessels, together with pericyte tubular or cord clusters [301]. It has been suggested that tumoral PCs originate endothelium-free vessel-like structures, that may play important, active and direct roles in tumor neoangiogenesis [87–92] (Fig. 8). An additional possible rationale for the demonstrated improvement of chemotherapy efficiency, in xenograft mouse glioma models after GBM-derived pericyte targeting [94], has given rise to the intriguing idea of identifying molecular markers for TNTs/MTs/TMs so as to pharmacologically disconnect the TNT/MT/TM communication networks [302]. This idea hypothesized the pericyte TNT/MT/TM-supported and pericyte-guided tumor angiogenesis roles in the control of cancer onset and progression.

### What do forebrain, retinal, mesenteric, and tumoral pericytes have in common?

NCSC-derived ectomesenchyme has been demonstrated to have a trophic effect on the early forebrain; in fact, the removal of the posterior diencephalic and mesencephalic neural folds produces massive cell death preceding the forebrain normal period of vascularisation [303]. When NCSCs migrate from the mesencephalic regions (midbrain) towards the forebrain, the forebrain is formed by a cranial telencephalon (“end-brain”) and a caudal diencephalon (“between brain”), which gives rise to optic cups. The latter is also surrounded by a layer of mesenchyme derived from NCCs. The wall of the optic cup is continuous with the neuroectoderm and will form the pigmented epithelium of the neural retina, while NCCs contribute to the stroma of the cornea, the ciliary and iris muscles, fibrous sclera, and vascular choroid layers, whose angioblasts are, however, formed by the mesoderm. It therefore seems conceivable that retinal capillaries have a composite origin, namely mesoderm-derived ECs and ectomesenchyme-derived PCs, and a PC-driven angiogenesis as described in the human cerebral cortex [86, 89, 304].

Nehls et al. [292] were the first to challenge the dogma of PCs as cells secondarily recruited to stabilize the newly-formed microvessel, and without any obvious role during the initial phase of vessel sprouting. In their study they investigated the angiogenic reaction of PCs, after intraperitoneal application of angiogenic stimuli utilizing whole-mount preparations of rat mesenteries and desmin immunocytochemistry. Their results show that PCs are involved in the earliest stages of capillary sprouting (see above) [292]. In this regard, and according to Sehgal [305], the enteric nervous system (ENS) predominantly originates from the vagal NCCs, located in an area between the brain and the spinal cord (post-otic hindbrain). From this area, NCCs migrate along dorsolateral and ventromedial pathways, through which this latter group enter the proximal foregut to give rise to the ENS. Once intrinsic ENS NCCs reach the foregut, they are referred to as enteric neural crest-derived cells (ENCCs). The classical theory is that ENCCs undergo unimodal rostral-to-caudal migration within the gut mesenchyme to colonize the entire length of the gut. This theory is now being challenged by alternative models envisaging a trans-mesenteric migration of NCCs. Using time-lapse imaging analyses of mouse ENCCs, Nishiyama et al. [306] captured an ENCC population that crosses from the midgut to the hindgut via the mesentery during a developmental time period in which these gut regions are transiently juxtaposed. They proposed that such ‘trans-mesenteric’ ENCCs constitute a large part of the hindgut ENS. It is conceivable that during their migration, ENCCs contribute to mesentery vascularization, living behind ‘angiogenic’ PCs that, together with ECs of the common splanchnopleuric mesoderm, form composite vessels with a dual origin.

Interestingly, more than half of all the GBM microvessel PCs have a host origin from endogenous brain PCs, rather than from tumor stem cells and/or bone marrow progenitors. Recent findings obtained in a GL261 mouse glioma model, orthotopically implanted in mice, demonstrate that much of the tumor pericyte population is contributed by PDGFR-β^+^/NG2^+^ re-activated PCs of the host cerebral cortex overlying the tumor [93]. Host brain-derived PCs have been identified as type-2, a pericyte subset that participates in normal angiogenesis and, when activated by the tumor, develops a strong tumor tropism. These PCs are integrated within the tumor vessels, and show specific angiogenic competence, being capable of inducing new vessel formation [102]. Overall, these data support the idea that NCC-derived forebrain PCs and their intrinsic angiogenic activity, displayed during human neocortex development, may spark neo-angiogenesis in both tumors and neurological diseases [103] (Table 1).

Finally, during chick NCC migration in living embryos, the presence of dynamic TNTs, involved in inter-NCC communication and cytoplasmic exchange, has been revealed [307], further supporting these cell structures as the common trait between forebrain, retinal, mesenteric and glioma PCs and their embryonic ancestors. Like in NCCs, PC-derived TNTs/MTs described in human cerebral cortex and in GBM may convey pro-angiogenic molecules, thus restricting the range of dispersion of spatial information and/or amplifying local signals in physiological and pathological vessel growth and collateralization [87].

## NG2 proteoglycan: a switch-on–off molecule involved in pericytes-driven angiogenesis

PCs are adept at receiving external signaling, migrating and rapidly adapting to achieve functional tasks, that include duplication and differentiation, in virtue of their extraordinary pluripotentiality [38–42, 68, 246, 265, 308]. This important capacity is determined by the expression of molecules able to sense and capture signaling molecules released from the surrounding environment. One of these molecules is proteoglycan NG2, a single-pass, type I transmembrane proteoglycan [274, 275]. The NG2 protein core is composed of a large extracellular domain (290 kD), carrying two to three glycosaminoglycan chains and a number of potential *N*-glycosylation sites, a single transmembrane tract, and a short cytoplasmic tail (8.5 kD) [309]. Nonetheless, NG2 can be expressed without chondroitin sulphate glycosaminoglycan chains, placing NG2 in the category of so-called part-time proteoglycans, specifically committed to bind, through the central domain of the core protein, basal lamina molecules [310–312] and a number of growth factors [274, 275, 311]. The involvement of NG2 in NVU/BBB organization has been demonstrated in vitro, where NG2 knockdown in PCs co-cultured with ECs reduces the endothelial barrier function [118] and in vivo in NG2-knock out mice, that show a modified arrangement of endothelial tight junction strands in cerebral cortex microvessels [10]. Even though NG2 displays little capacity for independent signal transduction, it is actually a regulator of cell surface domains and growth factor activities [275, 313]. In addition, working as a type I membrane protein, NG2 is subject to intramembrane proteolysis (RIP) regulated by α- and γ-secretases. The product of endogenous α-secretase action is the release of the NG2 ectodomain into the extracellular matrix. This process is termed shedding of soluble NG2 (sNG2) fragments [314–320]; four NG2 fragments have been associated with different biological functions in the CNS [321, 322]. The remaining C-terminal fragment undergoes a subsequent cleavage by γ-secretase, with the formation of an intracellular functional peptide, termed the released intracellular domain. The variety of NG2 and sNG2 biological roles has been investigated in NG2^+^ OPCs, where NG2 is maintained in mitotic active cells [323, 324] and is gradually downregulated until it disappears at the end of cell differentiation [325]. NG2 regulates cell motility via Rho/GTPase and polarity complex proteins [326] and has neuroprotective effects [327]. NG2 shedding from the OPC surface modulates the neuronal network and, in NG2 knock out mice, those neurons surrounding OPCs exhibit diminished AMPA (α-amino-3-hydroxy-5-methyl-4-isoxazolepropionic acid) and NMDA (N-methyl-D-aspartate) receptor-dependent current amplitudes [316, 322]. Interestingly, in the adult brain, NG2^+^ OPCs (also referred to as NG2-glia) contact neurons at axonal nodes of Ranvier and, in close proximity to synapses at neuronal cell bodies, express ion channels [328–331]. They increase NG2 RIP after neuronal activity, producing a functional switch toward the cell cycle S phase, and also increasing protein mRNA translation into proteins by modulating mTOR signaling components [332]. These observations may be pertinent to other NG2 expressing cells, especially immature/activated PCs. In fact, shed NG2 has been demonstrated to promote angiogenesis and migration of ECs via binding of sNG2 to galectin-3 and α3β1 integrin on the ECs, demonstrating that pericyte-derived NG2 is an important factor in promoting EC migration and morphogenesis during the early stages of neovascularization [295]. These include the formation of pericyte TNTs/MTs or effective pericyte conduits during both normal brain vascularization and tumoral neo-vessel formation [87, 88] (Figs. 7, 8, and Additional file 1: Figure S1). Accordingly, a decreased level of NG2 has been measured in cerebrospinal fluids derived from patients affected by Alzheimer’s disease [333] and Lewy bodies dementia [113], where pericyte-altered clearance of amyloid impedes vascular integrity and endothelial regeneration [317, 334–336]. Endothelial regeneration is also tightly regulated by endothelial/pericyte contacts through the activation of Notch1 RIP in a bone morphogenetic protein receptor 2-dependent pathway [337], although the effect of pericytes NG2 RIP has not yet been reported.

## The CXCL12/CXCR4 axis is involved in NCSC-derived pericytes signaling

**Fig. 9:**
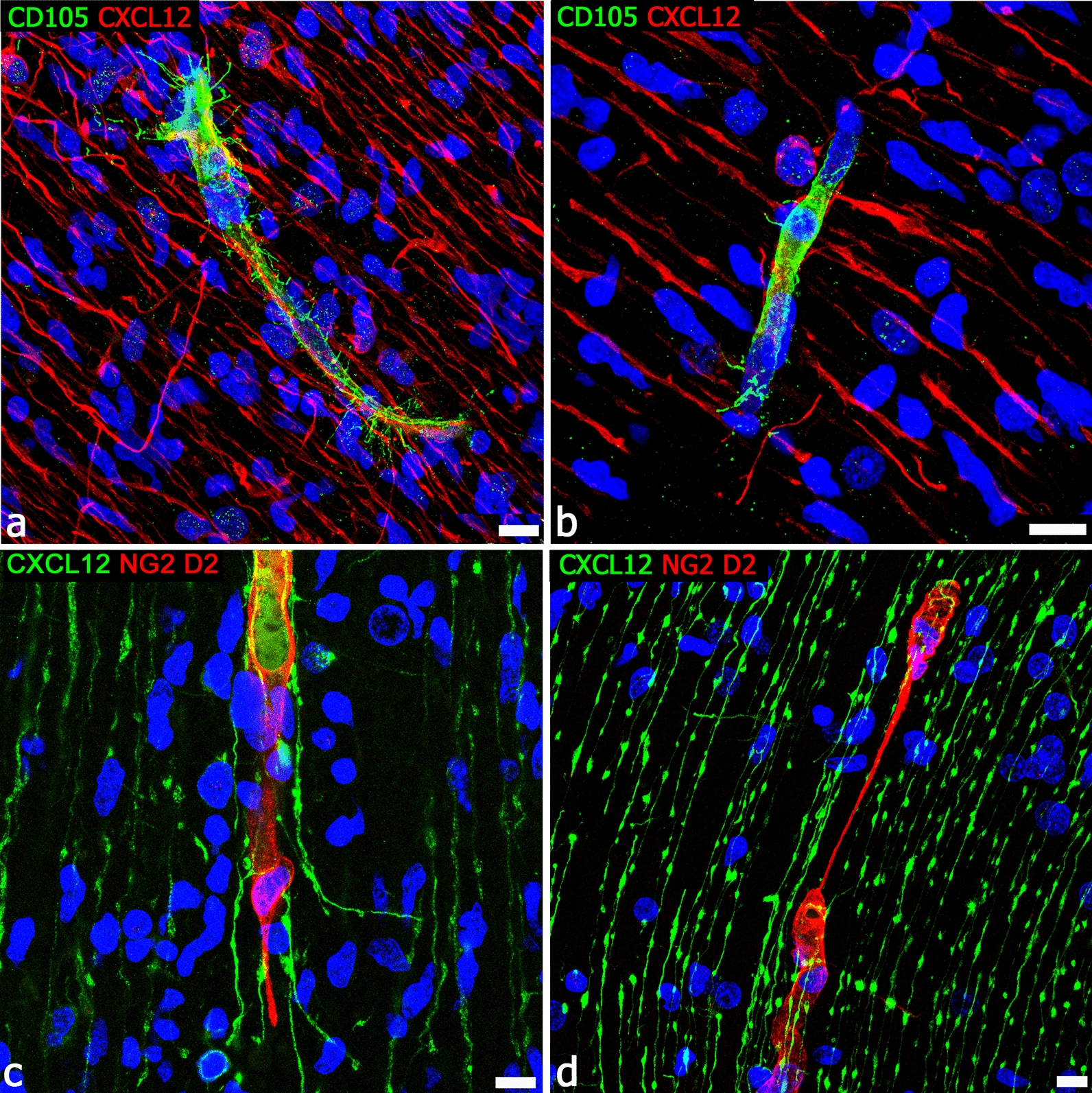
Interaction of chemokine CXCL12^+^ radial glia with endothelial sprouts and pericyte TNTs. **a**, **b** Typical CD105^+^ growing vessels, characterized by tip cells and filopodial processes, appear in extensive contact with CXCL12^+^ radial glia fibers. **c**, **d** NG2^+^ forebrain pericyte TNTs are contacted by CXCL12^+^ radial glia fibers. Human telencephalon 22 weeks of gestation. Scale bars **a** 25 µm; **b** 10 µm

**Fig. 10:**
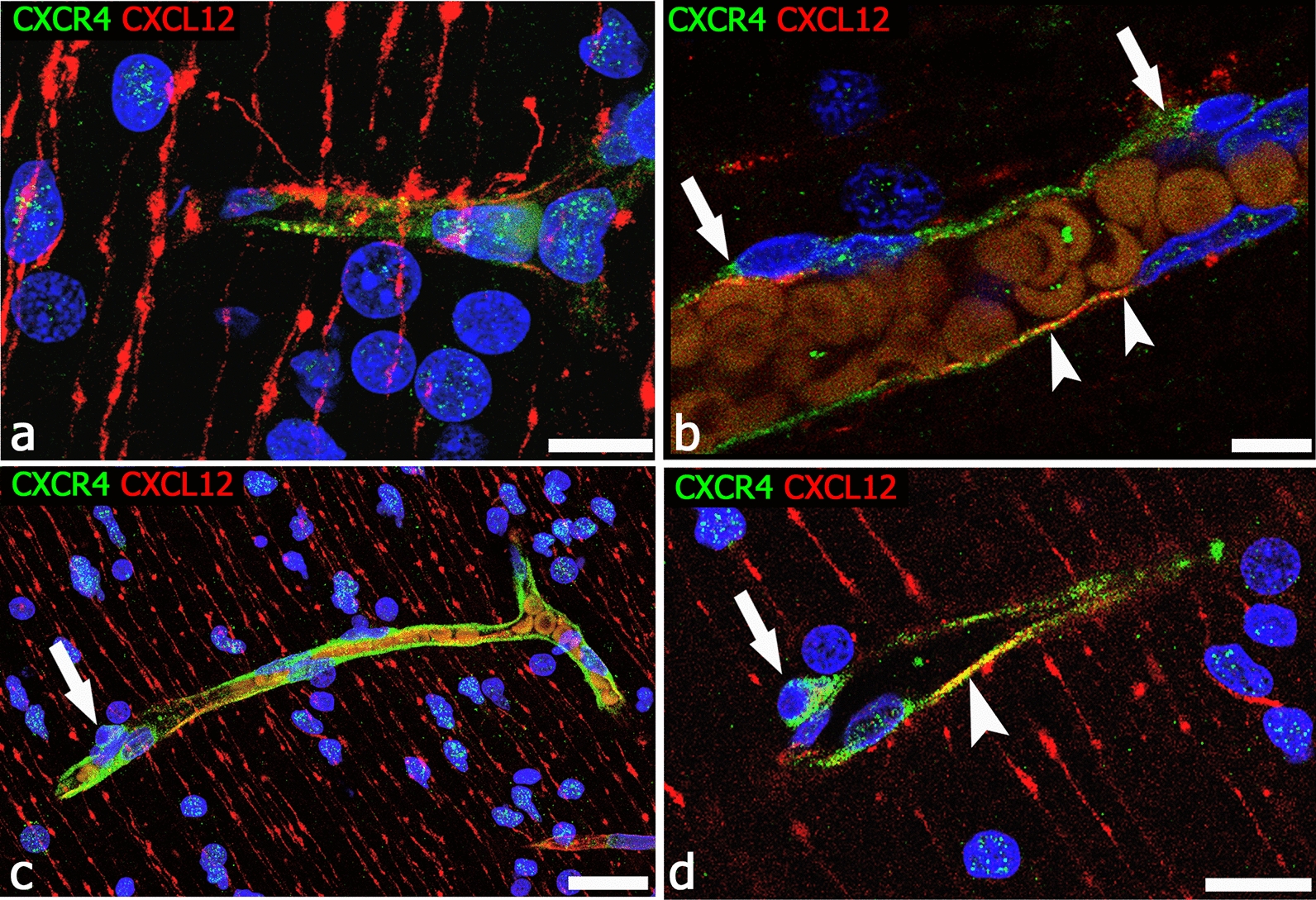
CXCL12/CXCR4 ligand receptor system interaction on ECs and PCs. **a** Radial glia stains for CXCL12 and forms extensive contacts with the CXCR4^+^ vessel wall; CXCR4 also marks neuroblasts nuclei. **b** CXCR4 reveals forebrain PCs (arrows), while CXCL12 is prevalent on ECs (arrowheads). **c** CXCR4 stains the wall of a vessel collateral and its PCs (arrow). **d** Enlargement of the pericyte shown in **c** (arrow) and a tract of CXCL12/CXCR4 colocalization on the vessel wall (arrowhead). CXCR4 nuclear expression in neuroblasts is the hallmark of their activated phenotype [346, 347]. Human telencephalon 22 weeks of gestation. Scale bars **a**, **b** 10 µm; **c**, **d** 25 µm

The expression of CXCR4 (chemokine C-X-C motif receptor 4) by forebrain PCs during vessel sprout formation is coincident with the demonstrated role of chemokine signaling in NCC migration. Chemokine CXCL12 (C-X-C motif chemokine ligand 12 or stromal cell-derived factor 1, SDF-1) and its cognate receptors CXCR4 and CXCR7 (chemokine C-X-C motif receptor 7) have been implicated in the regulation of cell migration in a variety of tissues and conditions, also during human brain neurogenesis and vascularization [181]. CXCR4 is required for the migration of many stem cell and progenitor cell populations from their respective niches to the differentiating tissues and organs, and it has been identified as a key component for NCC migration [338]. In addition, specific CXCR4 antagonists (AMD3100 and TN14003) disrupt the migration of mesencephalic NCCs, suggesting a role for CXCL12/CXCR4 signaling in the directed migration of mesencephalic NCCs in the early embryonic stages [339, 340]. The first, penetrating microvessels are followed by further waves of radial vessels that also elongate to parallel the progressively increasing width of the neural wall. At this time, typical endothelial sprouts coexist with a variety of forebrain PC-driven angiogenesis-associated structures [84] and, together with the classical signaling systems [127], alternative pathways, such as the CXCL12/CXCR4/CXCR7 ligand receptors systems, are involved in radial glia-like stem cells-microvessel and endothelial-pericyte interactions that are also seen to include pericyte TNT/MT structures (Fig. 9). In particular, in the developing cerebral cortex, chemokine CXCL12 is highly expressed by radial glia-like stem cells, immature radial astrocytes, perivascular astrocyte endfeet, and activated, CD105^+^ endothelial tip cells, while CXCR4 appears to be specifically expressed by sprout-associated PCs and migrating neuroblasts [180, 181] (Figs. 9, 10).

## Conclusions

An ample heterogeneity has been reported in PCs even in the same organs [71]; for example, brain PCs have distinct morphologies, markers, and functions along the arteriole–capillary–venule vascular bed [70]. In addition, PCs can have a heterogeneous origin, even within the same tissue. In the embryo, the forebrain (telencephalon and diencephalon) is the only part of the developing CNS into which mesencephalic NCCs penetrate, giving origin to a subpopulation of forebrain PCs. During human neocortex development and vascularization, NCC-derived, activated forebrain PCs are present as early as mitotic ECs, and almost completely ensheath the endothelial lining, forming de facto a tube-within-a-tube bi-layered vessel wall and participating in the very early steps of cortex angiogenesis. In the cortex, forebrain PCs give origin to TNTs, MTs, and autonomous conduits and leading sprouts, their state of angiogenic activation being always marked by the expression of proteoglycan NG2, adhesion molecule CD146, and chemokine receptor CXCR4. Proteoglycan NG2, also known as ‘high molecular weight melanoma-associated antigen’ (HMW-MAA), is also expressed by NCC-derived melanocytes, while the other two molecules are expressed by migrating NCCs and, according to our results, are still expressed by forebrain PCs (Figs. 5, 10).

Forebrain PCs may perform better than other CNS PCs in maintaining the BBB endothelial phenotype, stabilizing EC cord formation ‘in vitro’ [266, 341] and inducing barrier properties in primary and hematopoietic stem cell–derived ECs [259, 342, 343]. PCs denoted as ‘forebrain PCs’ are critical regulators of EC functions, including cerebral blood-flow and BBB regulation, as well as tube-formation. Models that recapitulate forebrain PCs in vivo ontogeny, by deriving them from hiPSCs in vitro via a neural crest intermediate, showed a cellular, behavioral and functional equivalence to in vitro-derived and in vivo-isolated normal, human forebrain PCs. This equivalence was demonstrated by cell migration and contractility assays and by the expression of genes associated with PC-specific biological processes, such as vesicular transport, formation, organization, and interaction of extracellular matrix, cell migration, contractility and angiogenesis [344]. hiPSCs can generate mesodermal cells and NCCs that can be induced to form mesoderm- and NCC-derived subpopulations of PCs, that specifically express the mesodermal genes, MIXL1 and TBXT, and NCCs genes, PAX3, PAX7 and TFAP2A [82, 345]. These findings promise to propel further investigation of specific roles of forebrain PCs, especially angiogenic properties, which are not yet fully understood. Accordingly, it will be crucial to explore transcriptional or epigenetic landscapes of forebrain PCs during angiogenesis, and neurovascular barrier properties *in vivo*, in vitro, and in different CNS diseases. The availability of single-cell RNA sequencing approaches, coupled with both genetic and pharmacological perturbations of forebrain PCs, makes it possible to identify signaling pathways that are triggered in the endothelial-forebrain PCs crosstalk to modulate angiogenesis and barriergenesis under such different conditions. A better knowledge of the ontogenetic PCs subpopulations may help to understand specific interactions and mechanisms involved in pericyte function/dysfunction, including normal and pathological angiogenesis, thereby offering an alternative perspective on cell subtype-specific therapeutic approaches. These studies could not only strengthen our understanding of the complex mechanisms involved in aberrant/tumoral vessel growth, but also provide us with new avenues for managing neurological diseases that could recognize angiogenic PCs as concurrent effectors in NVU ‘microvasculopathy’, suggesting therapeutic approaches that target both endothelial and the NCC/forebrain PC-specific angiogenic phenotypes and genotypes.

## Supplementary information

**Additional file 1: Figure S1.** The sequence of 34 single optical planes, from a z-stack image double stained with the endothelial marker CD31 (green) and the pericyte marker NG2 (red), shows a growing microvessel formed by a leading pericyte-derived endothelialized conduit. Human telencephalon 22 weeks of gestation. Original magnification 60×.

**Additional file 2: Figure S2.** Transmission electron microscopy images of newly-formed vessels in developing chick embryo brain. **a** A non–lumenalized microvessel with a continuous pericyte coverage (*arrowheads*), with few short projections toward the neuropil (*arrows*). **b**, **c** Small, lumenalized microvessels ensheathed by PCs (*arrowheads*). (from [5] with permission). Scale bars **a**, **b**, **c** 3 µm.

## Data Availability

Not applicable.

## Abbreviations

AMPA: α-Amino-3-hydroxy-5-methyl-4-isoxazolepropionic acid
BBB: Blood–brain barrier
CNS: Central nervous system
EC: Endothelial cell
ENCCs: Enteric neural crest-derived Cells
ENS: Enteric nervous system
GBM: Glioblastoma multiforme
Glut1: Glucose transporter isoform 1
hiPSC: Human induced pluripotent stem cell
MMP: Matrix metalloproteinase
MT: Microtube
mTOR: Mammalian target of rapamycin
NCC: Neural-crest cells
NCSC: Neural-crest stem cell
NG2: Neuron-glial antigen 2
NMDA: *N*-methyl-d-aspartate
NVU: Neuro-vascular unit
OPC: Oligodendrocyte precursors cell
PDGF-B: Platelet-derived growth factor-B
PDGFR-β: Platelet-derived growth factor receptor-β
PC: Pericyte
RIP: Regulated intramembrane proteolysis
sNG2: Soluble NG2
TGF-β: Transforming growth factor-β
TM: Tumor microtube
TNF-α: Tumor necrosis factor-α
TNTs: Tunneling nanotubes
VEGF: Vascular endothelial growth factor
VEGFR 2: Vascular endothelial growth factor receptor 2
