# Supplementary figures and images for: Neural crest cell-derived pericytes act as pro-angiogenic cells in human neocortex development and gliomas
Source: Fluids Barriers CNS. 2021 Mar 20;18:14. doi: 10.1186/s12987-021-00242-7 (PMC7980348; doi:10.1186/s12987-021-00242-7)

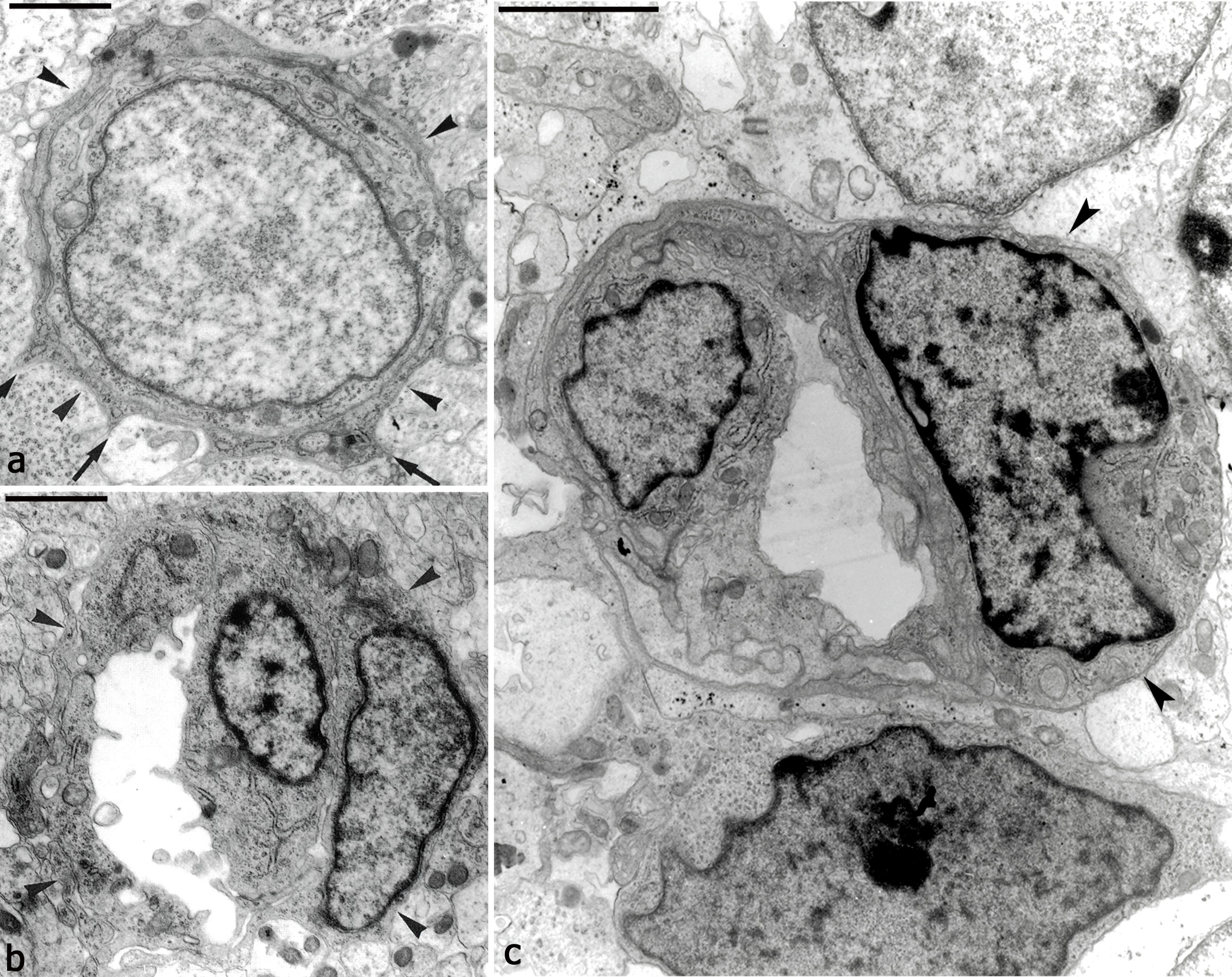

Supplement: Supplementary file 2 — Additional file 2: Figure S2. Transmission electron microscopy images of newly-formed vessels in developing chick embryo brain. a A non–lumenalized microvessel with a continuous pericyte coverage (arrowheads), with few short projections toward the neuropil (arrows). b, c Small, lumenalized microvessels ensheathed by PCs (arrowheads). (from [5] with permission). Scale bars a, b, c 3 µm. [file 12987_2021_242_MOESM2_ESM.tif]
